# Uncertainties in exposure predictions arising from point measurements of carbon dioxide in classroom environments

**DOI:** 10.1098/rsif.2024.0270

**Published:** 2024-10-23

**Authors:** Carolanne V. M. Vouriot, Maarten van Reeuwijk, Henry C. Burridge

**Affiliations:** ^1^ School of Mechanical, Aerospace and Civil Engineering, University of Sheffield, Mappin Street, Sheffield S1 3JD, UK; ^2^ Department of Applied Mathematics and Theoretical Physics, Centre for Mathematical Sciences, University of Cambridge, Wilberforce Road, Cambridge CB3 0WA, UK; ^3^ Department of Civil and Environmental Engineering, Skempton Building, South Kensington Campus, Imperial College London, London SW7 2BX, UK

**Keywords:** airborne infection, ventilation, risk modelling, computational fluid dynamics, carbon dioxide

## Abstract

Predictions of airborne infection risk can be made based on the fraction of rebreathed air inferred from point measurements of carbon dioxide (CO_${,}_{2}$_). We investigate the extent to which environmental factors, particularly spatial variations due to the ventilation provision, affect the uncertainty in these predictions. Spatial variations are expected to be especially problematic in naturally ventilated spaces, which include the majority of classrooms in the UK. An idealized classroom, broadly representative of the physics of (buoyancy-driven) displacement ventilation, is examined using computational fluid dynamics, with different ventilation configurations. Passive tracers are used to model both the CO_${,}_{2}$_ generated by all 32 occupants and the breath of a single infectious individual (located in nine different regions). The distribution of infected breath is shown to depend strongly on the distance from the release location but is also affected by the pattern of the ventilating flow, including the presence of stagnating regions. However, far-field exposure predictions based on single point measurements of CO_${,}_{2}$_ within the breathing zone are shown to rarely differ from the actual exposure to infected breath by more than a factor of two—we argue this uncertainty is small compared with other uncertainties inherent in modelling airborne infection risk.

## 1. Introduction

Since the work of Rudnick & Milton [1], carbon dioxide (CO_${,}_{2}$_) measurements have been used to infer the fraction of rebreathed air within indoor spaces and employed to assess the risk of far-field airborne infection therein. The COVID-19 pandemic provided a renewed focus on assessing airborne infection, and, as a consequence, CO_${,}_{2}$_ sensors have been deployed to many indoor spaces, especially within classrooms, both to help assess or manage the ventilation provision and to identify spaces at heightened risk. Herein, we quantify the uncertainties in potential infection exposures due to spatial variations within the indoor environment when exploiting point measurements of CO_${,}_{2}$_.

Airborne infection occurs when small droplets or aerosols carrying viral particles are emitted by an infectious individual, spread across a space on indoor air currents and are then inhaled by a susceptible individual. Unlike the other routes of transmission (e.g. infection via exposure to the spray of droplets at close range, or the contact route) airborne transmission can occur over large distances within an indoor space as well as between indoor spaces or buildings [2]. Measures such as social distancing or hand washing are relatively ineffective against the airborne route; instead, the airborne transmission of COVID-19 can be reduced through the use of engineering controls like ventilation or filtration [3]. Ventilation (i.e. the provision of outdoor air that refreshes the air indoors) is also essential for the mitigation of other airborne respiratory diseases [4] and crucial to providing comfortable and healthy environments for occupants, including being a key factor of indoor air quality.

Riley *et al*. [5] first presented the, now well-established, ‘Wells–Riley’ model of airborne infection risk, successfully applying this model to study a measles outbreak in a school. Underpinning the Wells–Riley approach is the concept of a ‘quantum’ of infection, as defined by Wells [6], which quantifies the number of infectious particles required to cause an infection. Appropriate quanta generation rates have been deduced for various respiratory infections, typically via retrospective analysis of outbreaks in operational environments. As such, the Wells–Riley approach (via quanta generation rates inferred from outbreaks) implicitly accounts for certain factors such as biological decay and deposition of the physical virus, which influence airborne transmission [7]. Although these factors can be more explicitly modelled, for instance by using a dose–response approach to airborne infection risk modelling, other challenges then arise when individually parametrizing the exposure dose of the virus and the physiological response of occupants. As such, the Wells–Riley model remains widely used to study the far-field airborne transmission of a range of diseases [8].

Typically, the Wells–Riley model requires an estimate of the ventilation provision to a given space, which can be challenging to measure practically. An alternative has been to use the rebreathed fraction of air to more directly estimate the risk of airborne infection [1]. The rebreathed fraction is the fraction of the air within a space that has already been exhaled by an occupant (some fraction of which could be infected due to the presence of infectious individuals)—crucially, the rebreathed fraction is readily assessed via the measurements of the carbon dioxide (CO_${,}_{2}$_) concentration within indoor air. From Rudnick & Milton [1], the probability of infection $P_{A}$ when a space is occupied by infectious and susceptible individuals is given by


(1.1) $$P_{A}=1-\exp\;\left(-\int_{0}^{T_{A}}\frac{I}{N}\;f\;q\;dt\right),$$

with I being the number of infectious individuals, N the total number of occupants, q the quanta generation rate, $T_{A}$ the period under consideration and $f=(C-C_{a})/C_{p}$ the rebreathed fraction (C(x,y,z) is the CO_${,}_{2}$_ concentration measured at a point (x,y,z) within the space, $C_{a}$ indicates the outdoor ambient level and $C_{p}$ is the concentration of CO_${,}_{2}$_ added through exhaled breath).

This method has been widely used in school classrooms [9–11], where CO_${,}_{2}$_ monitoring is becoming widespread, in part as a response to the COVID-19 pandemic. Classrooms are densely occupied spaces where the general occupancy patterns are known, and as such CO_${,}_{2}$_ is generally deemed to be a reasonable proxy for ventilation. In addition, schools, and classrooms in particular, are a crucial setting where airborne transmission needs to be better understood and mitigated, since, in the UK alone, 11 million people attend schools daily and much of this time is spent indoors, often in classrooms. CO_2_ measurements, therefore, seem to offer a way to assess the ventilation provision and risk of airborne infection at a reasonable cost and at a large scale. However, when calculating the risk of airborne infection via these methods, it is often necessary to assume that a single point measurement of CO_${,}_{2}$_ is representative of the entire space, implicitly assuming that CO_${,}_{2}$_ and quanta are well mixed throughout the space. The full implications of this assumption in this context are not well evidenced; it is expected that naturally ventilated spaces (which constitute the majority of classrooms in the UK) might be a particular challenge as they are spaces where mixing within the room and airflow patterns are likely to be more variable. It is valuable to determine what the impact of this assumption is on the assessment of airborne risk infection, taking naturally ventilated classrooms as an example. In this article, data from the numerical simulations of an idealized naturally ventilated classroom (see Vouriot *et al.* [12] for validation) are exploited to compare the distribution of CO_${,}_{2}$_ concentrations within the room with that of the breath of an infectious occupant. This work focuses on the prediction of the far-field exposure, which is often used as an input to airborne risk models, with the aim of understanding the uncertainty introduced by using CO_${,}_{2}$_ as a proxy and how this varies under different environmental conditions. Details on the flow field established and the resulting overall CO_${,}_{2}$_ distribution are given by Vouriot *et al.* in [12] and [13], respectively.

The aim of this article is to deploy a new method on existing computational fluid dynamics (CFD) data representing idealized naturally ventilated classrooms. The accuracy of the well-mixed assumption underpinning the analysis of Rudnick & Milton [1] can then be evaluated and the uncertainties introduced by using a limited number of CO_${,}_{2}$_ measurements to assess airborne infection risk can also be investigated. We exploit three-dimensional measurement fields obtained via numerical simulations to track two different tracers. One represents the carbon dioxide exhaled by all occupants in the room while the other represents the infected breath of a specific individual. The location of the infectious occupant is varied along with the ventilation configuration and flow rate. The methodology is introduced in §2, and results are presented in §3. Finally, conclusions are drawn in §4.

## 2. Methodology

A series of CFD simulations are carried out to study the distribution of infected breath originating from a single infectious individual in an idealized naturally ventilated UK classroom. CFD is chosen because it allows the assessment of the impact of the ventilation system on contaminant distribution within a room. In addition, since the full three-dimensional fields can be investigated, the distribution of a tracer representative of the infected breath can be directly compared with a tracer representing the overall CO_${,}_{2}$_ field generated by all occupants. Focus is drawn to far-field exposure since the simulations are intended to represent an idealized scenario and do not capture the close range dynamics.

The simulations are conducted with the open source CFD code OpenFOAM v. 2106 using the transient buoyant­Pimple­Foam solver. A Reynolds-averaged Navier–Stokes (RANS) turbulence model is used, the k−ω shear stress transport (SST) model, which is expected to capture the main features of ventilating flows. In addition, Vouriot *et al.* [12] compare the flow patterns obtained with this set-up with small-scale experiments and show they display comparable features, including a distinctive horizontal convective pattern. Further details on this set-up and the validation process are also given by Vouriot *et al.* [13,14].

**Figure 1.**
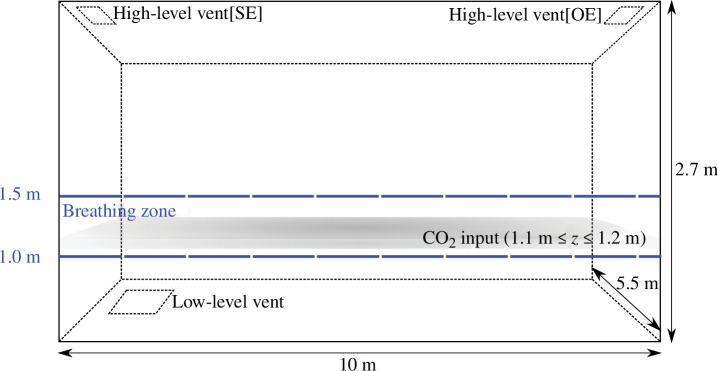
Illustration of the set-up used to represent a generic naturally ventilated UK classroom (either in a single-ended (SE) or opposite-ended (OE) configuration). The heat is input on the floor. A passive scalar representing CO_${,}_{2}$_ is introduced between the heights of 1.1 and 1.2 m. Exposure is assessed in the breathing zone shown in blue.

A classroom domain of size 10 × 5.5 × 2.7 m, is considered, as shown in figure 1. It is connected to larger exterior boxes through low- and high-level vents that both lay in horizontal planes, a set-up often used to represent idealized spaces with buoyancy-driven displacement ventilation [15]. Overall, close to 1.4 million hexahedral grid cells are used, with overall simulations consuming around 15 000 cpu hours. The ventilating flow is driven solely by buoyancy in this scenario and is set here by imposing a 6200 W distributed heat flux on the classroom floor. This heat input corresponds to the heat generated by 32 occupants, which is typical in UK classrooms [16], along with the heat provision required to provide a thermally comfortable environment. A wintertime scenario is investigated where the ambient outdoor temperature is set to $T_{a}=278$ K (5°C), which is taken to be typical for the coldest months in the UK. The focus is drawn to the heating season as it corresponds to the period when ventilation might be limited and thus infection risk might be the highest [11].

Two ventilation configurations are investigated to study the impact of the vent location on the ventilating flow and the exposure to infected breath. With all other parameters remaining the same, the location of the high-level vent, the classroom ventilation outlet, is modified leading to

— an ‘opposite-ended’ (OE) configuration in which the low- and high-level vents are at opposite ends of the classroom,
— a ‘single-ended’ (SE) configuration in which the low- and high-level openings are both at one end of the classroom.

The low-level vent has an area $A_{l}$ of 0.4 m^2^ and is located near the top left of the classroom horizontal cross section (figure 1). In both configurations, the high-level vent has an area $A_{h}$ equal to 0.2 m^2^. The position of the vent is altered from being located in the back-right for the opposite-ended configuration to being moved to the same end as the low-level opening and located in the back-left of the cross section in the single-ended configuration (figure 1).

This study is aimed at establishing the spatial variations in exposure that might occur over the relatively long durations that are relevant to airborne exposure and infection. As such, the simulations (although run using a transient solver for technical reasons as detailed by Vouriot [14]) are steadily forced, allowed to develop until a statistically steady state is reached and then run and averaged over durations representative of the typical exposure durations in classrooms [14]. Thus, there is no need to incorporate a specific model for each individual’s breathing, and occupants’ breath is modelled via a steady source of CO_${,}_{2}$_. The overall CO_${,}_{2}$_ generated by all occupants is represented by the addition of a passive non-buoyant scalar over the entire cross section of the classroom between the heights of 1.1≤z≤1.2 m, the typical breathing height of seated primary school pupils [16]. The average CO_${,}_{2}$_ generation rate of a pupil is 0.00335 l s^−1^ per person, which is taken to be typical of a primary school-aged (6–11 years old) child doing a relatively quiet activity, such as sitting at their desk [17]. For the 32 occupants considered in this study, this corresponds to an overall CO_${,}_{2}$_ generation rate of 0.1072 l s^−1^. In this work, the occupants are assumed to be uniformly distributed and the effect of a particular seating arrangement or the impact of a student/teaching desk arrangement is not assessed.

**Figure 2.**
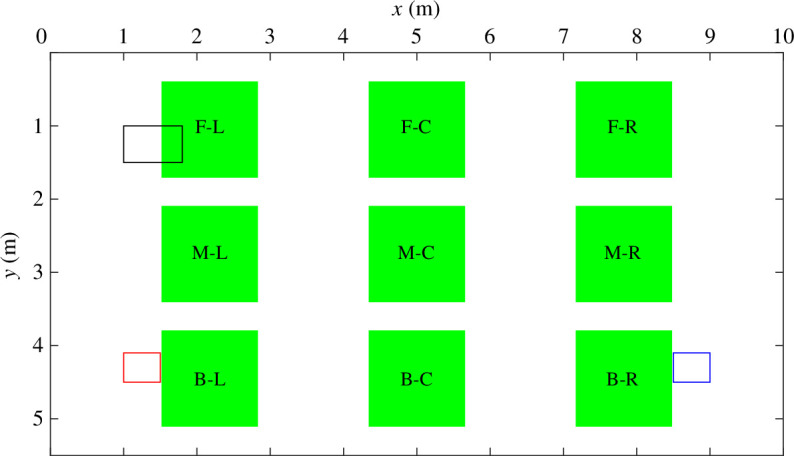
Classroom horizontal cross section. Scalar $B_{i}(x,y,z)$ represents the infected breath and is introduced at the nine different locations shown by the green areas between the heights of 1.1 and 1.2 m. The zones are labelled by splitting the room into front (F), middle (M) and back (B) rows and left (L), central (C) and right (R) columns. The black rectangle (towards the top left) shows the horizontal position of the inlet (located at floor height, z=0 m). The blue rectangle (towards the back-right) shows the position of the opposite-ended (OE) high-level vent and the red rectangle (towards the back-left) shows its position in the single-ended (SE) configuration, both located at ceiling height (z=2.7 m).

In addition, a further nine tracers are introduced to represent infected breath arising from specific infectious individuals located in any one of the nine locations. We describe these nine locations on a grid splitting the room into: front, middle and back rows; and left, central and right columns (as shown by the green areas in figure 2). A choice is made to introduce these tracers not at a point source—which would require a careful choice of a specific breathing model and validation that this model can achieve appropriate dispersion to the far field. Instead, an additional nine volume sources, each emitting a unique tracer, are added so that the ‘breath’ of an infected occupant is effectively dispersed over the portion of the room associated with that individual; this is taken to be the fraction of the breathing zone $S/N=1.7\;{\text{m}}^{2}\approx (5.5\times 10)/32$ m^2^. The use of passive tracers to represent infected breath implies that the natural decay of infected aerosols is not explicitly reflected. Therefore, the approach taken represents a mildly conservative limiting case in this regard (although highly variable, typical values for the half time of the viability of SARS-CoV-2 range from a couple to hundreds of minutes [18] whereas the air change rates in the simulations considered here are between four and nine air changes per hour). It should be noted that, due to the complexity of approximating the size distribution and composition of viral droplets, the uncertainty of a tracer gas model is likely to be of the same order of magnitude as uncertainties inherent within any other particular parametrization that could be deployed within more direct simulations of the droplets [2]. Crucially, Pei *et al.* [19] showed that passive tracers do suitably represent the dynamics of the small aerosols, which are likely to be responsible for the far-field airborne transmission investigated.

The nine unique tracers, which represent infected breath, were all introduced at once within the simulations for reasons of efficiency; for reasons of presentation, we describe them consecutively so that we consider only a single infectious individual being present at one of nine different locations, at any given time. Similarly to the passive scalar representing CO_${,}_{2}$_, these additional unique nine scalars are introduced between heights 1.1≤z≤1.2 m, each with a generation rate of 0.00335 l s^−1^ that corresponds to an individual’s breathing flow rate in this set-up. This method allows the direct comparison of two scalars. The scalar C(x,y,z) represents the overall CO_${,}_{2}$_ concentration produced by all 32 occupants and corresponds to what is practically measured by CO_${,}_{2}$_ monitors. The scalar $B_{i}(x,y,z)$ represents the breath of an infectious individual located in region i, one of the nine locations investigated and highlighted in figure 2. The release of this scalar has been scaled and set up so that it is equal to the fraction of CO_${,}_{2}$_ exhaled by one of the classroom’s occupants. In this way, the contribution of a specific infectious individual can be identified and compared with the overall CO_${,}_{2}$_ concentration.

## 3. Results

### 3.1. Description of the flow field within the classroom

**Figure 3.**
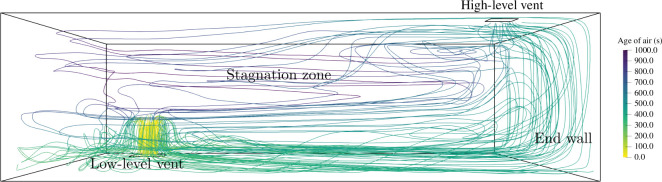
Flow field in the opposite-ended configuration for the original set-up. The streamlines originate from the low-level vent and are coloured by the age of air. The flow fields obtained with different ventilation configurations and flow rates are described in further detail by Vouriot *et al.* [13].

To understand the distribution of infected breath within the classroom, it is vital to understand the indoor air flows within which infected breath is transported and dispersed. The flow fields within the simulations presented have been thoroughly discussed by Vouriot *et al.* [12], with a complementary discussion of the CO_${,}_{2}$_ distribution presented by Vouriot *et al.* [13]. In summary, the ventilation rate is similar in both configurations, and close to 7.5 l s^−1^ per person; however, the position of the high-level vent significantly impacts the flow pattern in the classroom. This is evidenced by the contaminant removal efficiency that compares the room averaged CO_${,}_{2}$_ concentration with the theoretical concentration expected in a well-mixed room. In the opposite-ended configuration, it is close to unity, similar to the efficiency in a well-mixed space, whereas in the single-sided configuration it is 25% higher, closer to the efficiency of displacement ventilation. Figure 3 shows how a fluid parcel travels through the room in the opposite-ended configuration with streamlines originating from the low-level vent and coloured by the age of air, a measure of how fresh the air is. In both the opposite- and single-ended configurations, the flow established is similar in the lower half of the classroom with the incoming cold air flowing through the inlet and warming as it flows across the floor of the classroom before starting to rise along the end wall. In the opposite-ended case, the flow can then directly exit through the outlet, leaving a large region in the top left corner of the room with little ventilation where the flow can stagnate, generating higher residence times and CO_2_ concentrations. This is not the case in the single-ended case where the air continues to flow along the ceiling before reaching the high-level vent, therefore ventilating a larger proportion of the classroom and leading to an overall better contaminant removal efficiency.

### 3.2. Distribution of infected breath within the classroom’s breathing zone

The distribution of infected breath is analysed for each potential infectious individual location and for both the opposite-ended and single-ended configurations in the breathing zone. The resulting tracer concentration of infected breath released in location i is denoted $B_{i}(x,y,z)$. Releases in each one of the nine locations (shown in figure 2) are systematically investigated; for example, the resulting tracer concentration of infected breath released in the front-left location F-L is denoted $B_{F-L}(x,y,z)$. Each infected breath tracer is released with equivalent fluxes to that of CO_${,}_{2}$_ production within an individual’s breath. This enables the specific contribution of an individual to the overall CO_${,}_{2}$_ concentration to be assessed at any given location, thus determining how it varies with the location of the source (i.e. the position of the infectious occupant breathing out) and the ventilating flow patterns.

Since our focus is on infection risk, we seek quantities that might represent the concentrations occupants are exposed to; we, therefore, average a number of quantities over the vertical extent of the breathing zone. This is defined as the region between the heights $z_{0}=1$ m and $z_{0}+h_{\mathrm{bz}}=1.5$ m (which is expected to represent the seated and standing head heights of the classroom’s occupants). For instance, the average of the tracer concentration of infected breath released in location i over the vertical extent of the breathing zone, is defined as


(3.1) $$\langle B_{i}\rangle_{bz}\;(x,y)=\frac{1}{h_{bz}}\int_{z_{0}}^{z_{0}+h_{bz}}B_{i}\;(x,y,z)\;dz.$$

The average CO_2_ concentration within the entire breathing zone (i.e. over the volume, $S\times h_{\mathrm{bz}}$, where S is the horizontal cross-sectional area of the room) also proves to be a useful metric, which we define as


(3.2) $$\langle C\rangle_{bz,\;S}=\frac{1}{S}\int_{S}\left\{\frac{1}{h_{bz}}\int_{z_{0}}^{z_{0}+h_{bz}}C\;(x,y,z)\;dz\right\}dA.$$

Finally, we define the normalized concentration of the infected breath,


(3.3) $$\beta_{i}\;(x,y)=\frac{N\langle B_{i}\rangle_{bz}\;(x,y)}{\langle C\rangle_{bz,\;S}-C_{a}}\;,$$

where N is the number of occupants (here 32) and $C_{a}$ is the ambient CO_${,}_{2}$_ concentration (here taken to be 400 ppm).

**Figure 4.**
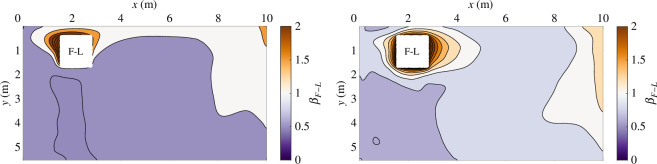
Horizontal cross section of the normalized infected breath scalar concentration originating from the front-left (F-L) location ($\beta_{F-L}$) for: (*a*) the opposite-ended and (*b*) the single-ended configuration. The white square represents the near-field region and is not considered in this work.

Figure 4 presents a map, across the room, of the normalized concentration of the breath from a single infected individual. Within the figure, we assume the infector is located at the front-left (F-L) of the room, so that figure 4 plots the data of $\beta_{F-L}$, for both ventilation configurations. The near-field region is shown by a white square and is not explicitly considered in this work. Regions where $\beta_{F-L}=1$ indicate that the concentration of the infected breath scalar is equal to the average concentration of rebreathed air within the breathing zone. In regions where $\beta_{F-L}>1$, the scalar originating from F-L contributes more to the overall measured CO_${,}_{2}$_ concentration than the average fraction. This is shown in orange colours in figure 4 and is often found close to the occupant’s location, as can be expected.

**Figure 5.**
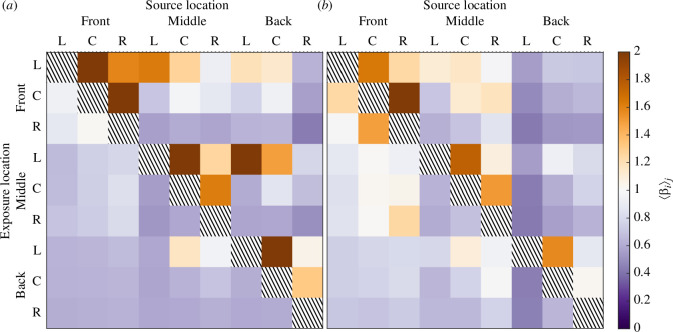
Normalized infected breath scalar concentration $\langle \beta_{i}\rangle_{j}$ in the zones shown in figure 2 for: (*a*) the opposite-ended (OE) configuration and (*b*) the single-ended (SE) configuration. The columns show the source of the scalar (the infectious individual location) and the rows the location where the concentration is measured (the exposure location). The hatched diagonals represent the near-field exposure and are not considered. In both configurations, the low-level vent is positioned below the front-left (F-L) zone. In the opposite-ended configuration (*a*), the high-level vent is located above the back-right (B-R) zone and above the back-left (B-L) zone for the single-ended case (*b*).


To illustrate the distribution of exposures arising throughout the room when an infected individual is located in each of the nine locations investigated, we create a nine-by-nine matrix (shown for each ventilation configuration in figure 5). The columns indicate a given source location i, and the rows show the remaining eight regions (j) where exposure is assessed. As we are not considering the near-field, exposure is not assessed at the source location (the diagonal of the matrix). To enable this, we define the average concentration, in region j, of the tracer of infected breath originating from location i, as

(3.4) $$\langle \beta_{i}\rangle_{j}=\frac{1}{A_{j}}\int_{A_{j}}\beta_{i}\;dA\;,$$

where $A_{j}$ is the area of region j. This corresponds to the area of one of the nine (green) regions illustrated in figure 2, which are intended to represent the input from one occupant and hence have an area set by S/N. For example, $\langle \beta_{F-L}\rangle_{F-R}$, the average value of $\beta_{F-L}$ at the front-right (F-R) of the room (shown at the top-right corner of figure 4) is shown in the first column, third row down of each exposure matrix.

Thus, figure 5 highlights source-location and exposure-location combinations where the concentration of infected breath is smaller than the average concentration of rebreathed air (measured from the CO_${,}_{2}$_ concentration) in violet to purple colours, and combinations that lead to relatively larger concentrations in orange to brown colours. The highest values are located close to the diagonal, demonstrating that, as expected, relatively high concentrations of infected breath are found in close proximity to the location of the infected breath source. However, in the opposite-ended configuration, shown in figure 5*a*, the highest normalized concentrations are found at exposure locations (as shown by the rows of the matrices) in the front row and left column (F-L) and middle row and left column (M-L) of the classroom cross section—these locations correspond to regions where the flow is relatively stagnant. The lowest normalized concentrations (shown in the purple spectrum) are calculated across exposure locations in the right column (F-R, M-R and B-R), near the classroom end wall, which is a region in which the fluid flow is predominately vertical and of relatively high velocity (see figure 3). The spread of the scalar remains limited if the release (as shown by the columns of the matrix) is located in the stagnating area (at the front-left (F-L) or middle-left (M-L) of the room) or near the outlet of the opposite-ended configuration (above the back-right (B-R) zone). Similarly, in the single-ended configuration the highest normalized concentrations are also found near the diagonal as well as at the front-left and front-centre (F-L and F-C), whereas low normalized values are calculated in the back-centre and back-right (B-C and B-R) (see figure 5*b*). However, the figure also shows that the values of normalized concentration are typically closer to unity with this configuration, and the exposure locations with $\langle \beta_{i}\rangle_{j}>1$ are sometimes different to those observed for the opposite-ended configuration—showing the impact of the different flow patterns on the distribution of scalar in the breathing zone. In the single-ended configuration, the scalar is more likely to spread if released in the centre of the classroom (M-C) and, once again, the scalar released closest to the high-level vent (in this case, at the back-left, B-L) will not spread significantly.

**Figure 6.**
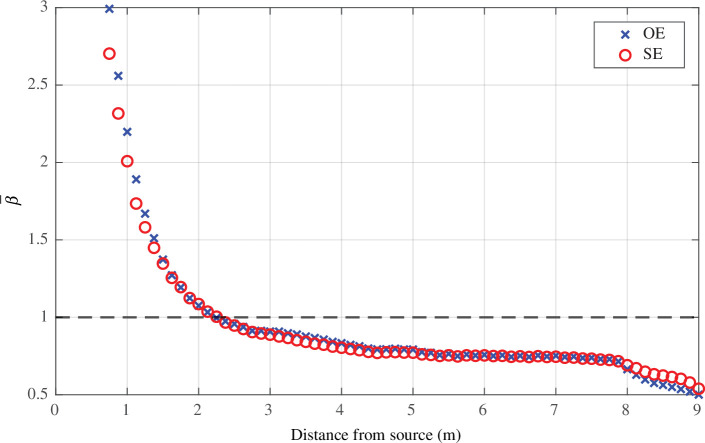
Mean normalized infected breath scalar concentration $\bar{\beta }$ with respect to the distance from the scalar source for the opposite-ended (OE) and single-ended (SE) configurations. This is averaged for all nine release locations. The horizontal dashed line shows $\bar{\beta }=1$.

For both configurations, higher normalized concentrations are often found near the diagonal of figure 5; i.e. not unexpectedly, concentrations are higher closer to the location of the infectious individual. This can be further illustrated by finding the mean normalized infected breath scalar concentration, averaged over the height of the breathing zone, at a given (radial) distance from the centre of the release location. This data can be analysed for each source location and we plot the average $\bar{\beta }(x,y)=1/9\sum_{i=1}^{9}\beta_{i}\;$, for each ventilation configuration in figure 6. In both cases, on average, the normalized scalar concentration quickly decays away from the source and drops below unity within 2 m of the release location. Due to the averaging performed, the local variations in normalized concentration are not captured in this figure, which instead highlights the general trend observed for all potential release locations.

### 3.3. Exposure estimates from local CO_2_ measurements

In this section, we address the extent to which CO_${,}_{2}$_ concentration can be used as a proxy for far-field exposure, which is then typically used as an input to airborne infection risk models.

When modelling the risk of airborne infection following equation (1.1), a CO_${,}_{2}$_ proxy exposure $E_{p}$ rate is used, with


(3.5) $$\begin{array}{ccc} & E_{p}=\frac{qI(C-C_{a})}{N\;C_{p}}\;, & \end{array}$$

where in the case considered here, following Vouriot *et al.* [11], the number of infectious occupants I is taken to be one and the classroom is assumed to be fully occupied with N the total number of occupants, here N=32. From our simulations, for any given scenario, the exposure rate calculated from the scalar representing infected breath $B_{i}(x,y,z)$ is


(3.6) $$\begin{array}{ccc} & E_{a}=\frac{qB_{i}}{C_{p}}\;. & \end{array}$$

The local ratio of the infected breath proxy to CO_${,}_{2}$_ proxy exposure ratio,


(3.7) $$\begin{array}{ccc} & R_{l}=\frac{E_{a}}{E_{p}}=\frac{B_{i}\;N}{I(C-C_{a})}\;, & \end{array}$$

gives a measure of whether the CO_${,}_{2}$_ concentration C(x,y,z) at a point is a suitable proxy to assess exposure to infected breath.

Crucially, the difference between $\beta_{i}$ and $R_{l}$ is that a high value of $\beta_{i}$ indicates regions where there is a greater potential for exposure to infected breath. On the other hand, $R_{l}$ is an indication of whether this potential for exposure can be captured by CO_${,}_{2}$_ point measurements. A low $R_{l}$ does not indicate that there is a low potential for exposure, but instead, that using CO_${,}_{2}$_ to estimate exposure is likely to lead to overestimates of the exposure, and thus by extension, overestimates of the risk of airborne infection.

**Figure 7.**
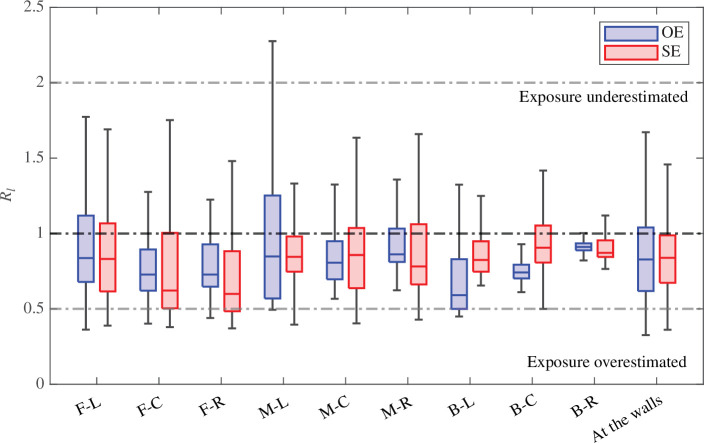
Distribution of the infected breath to CO_2_ proxy exposure ratio $R_{l}$ for all nine infectious individual locations for both the opposite-ended (OE) and single-ended (SE) configurations in the breathing zone (1≤z≤1.5 m). Measurements at the walls are defined as being located within 0.2 m of a wall. The bottom and top of the boxes show the 25th and 75th percentiles, the median is shown by the central line. The whiskers include data within one and a half of the interquartile range from the first and third quartiles. Horizontal dashed lines show a ratio equal to 0.5, 1 and 2, respectively.

Figure 7 shows the distribution of $R_{l}$ at different measurement locations for all potential locations of infectious individuals. For practicality, this includes the regions defined previously in figure 2 as well as a region near the classroom walls. This is defined as measurements taken with 0.2 m of a wall in the breathing zone and is of particular interest as this will be where measurements are often made in practice in classrooms. Figure 7 clearly shows there are variations in $R_{l}$, both between the two ventilation configurations and depending on where CO_${,}_{2}$_ is measured. For instance, the spread of values of $R_{l}$ observed in the middle-left (M-L) region is much larger than at the back-right (B-R) of the room for the opposite-ended configuration. The latter is a region where there is a strong ventilating current that is likely to strongly affect both the infected breath scalar and overall CO_${,}_{2}$_ (see [13] for more details) thus improving the predictions of exposure when using CO_${,}_{2}$_. The two scalars can be less correlated in the middle-left (M-L) region where there is a large stagnating zone in the opposite-ended configuration. Measurements taken at the walls are broadly within the range seen in other locations, which indicates that they are not particularly worse when it comes to estimating exposure and thus remain an appropriate measurement location.

Overall, almost all distributions are entirely contained between ratios of 0.5 and 2, which means that the infected breath to CO_${,}_{2}$_ proxy exposure ratio can, for both configurations, be considered accurate to within a factor of two. Exposure to infected breath is likely to remain high near the source and this can lead to underestimation when using a CO_${,}_{2}$_ proxy exposure and conversely, local increases in CO_${,}_{2}$_ concentration might lead to overestimates.

### 3.4. Sensitivity to changes in the flow rate

All other parameters remaining the same, the sensitivity of the previous results to changes in the ventilation provision is investigated by varying the areas of the high- and low-level vents. Details on the ventilation induced by each set-up are given by Vouriot *et al.* [13] and a summary of the four additional simulations performed for each configuration is included in table 1. The resulting ventilation rates vary by a factor of two between the different simulations with considerable differences in the ventilating flow patterns. This is indicated by contaminant removal efficiencies ranging from 0.86, evidencing short-circuiting, to 1.32, corresponding to the efficiency of displacement ventilation, giving effective ventilation rates ηQ that vary by a factor of three between the simulations. Overall, the ventilation provision per occupant varies between 4.81 and 11.52 l s^−1^ per person which, although significant, remains within the range measured in classrooms [20,21].

**Table 1.** List of simulations performed. The simulations described previously correspond to the original vent set-up (OS). $A_{h}/A_{l}$ is the vent area ratio, $Q_{p}$ is the per-person ventilation flow rate obtained in each scenario and η is a measure of the contaminant removal efficiency, given for both the opposite-ended (OE) and single-ended (SE) configurations.

case	description	$A_{h}/A_{l}$	vents	$Q_{p}$ (l s^−1^/person)	η
OS	original set-up	0.50	OE	7.47	1.05
			SE	7.54	1.24
SO	smaller openings	0.50	OE	4.81	1.01
			SE	4.81	1.23
SOR	smaller openings and vent area ratio	0.25	OE	5.15	0.86
			SE	5.15	1.19
LO	larger openings	0.50	OE	11.45	1.05
			SE	11.52	1.18
LOR	larger openings and vent area ratio	2.00	OE	11.01	1.24
			SE	11.16	1.32

**Figure 8.**
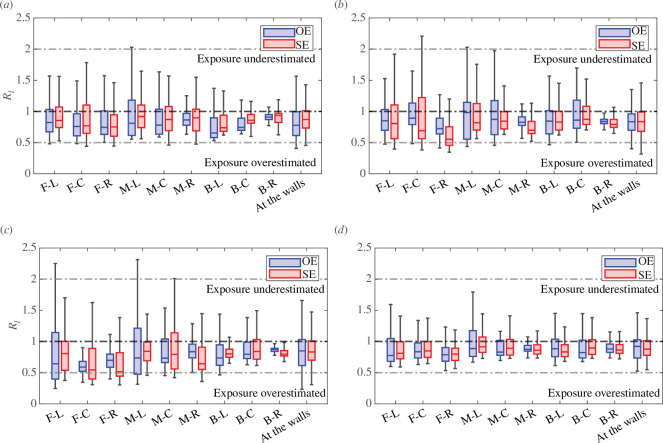
Distribution of the infected breath to CO_2_ proxy exposure ratio $R_{l}$ for all nine infectious individual locations in the breathing zone (1≤z≤1.5 m) for both the opposite-ended (OE) and single-ended (SE) configurations for the set-ups described in table 1. The bottom and top of the boxes show the 25th and 75th percentiles, the median is shown by the central line. The whiskers include data within one and a half of the interquartile range from the first and third quartiles. Horizontal dashed lines show a ratio equal to 0.5, 1 and 2, respectively. A similar distribution for the original vent set-up (OS) is shown in figure 7.

The distributions of the infected breath to CO_${,}_{2}$_ proxy exposure ratio $R_{l}$ are shown for all scenarios considered in figure 8. Despite significant changes in the ventilation provision, the trends remain similar to what is observed for the original vent set-up (OS) presented in figure 7. This also includes the decay of the normalized scalar concentration, shown in figure 6 for the OS set-up, with all scenarios similarly showing that $\bar{\beta }$ drops below unity within 2 m of the release location. Across the breathing zone, for all potential release locations of infected breath scalars considered, the distributions of $R_{l}$ have a median close to unity and interquartile ranges between 0.8 and 1.5. In all cases, the exposure predicted from local CO_${,}_{2}$_ measurements is within a factor of two of the exposure to the infected breath scalar in the majority of the breathing zone and the differences between the opposite-ended and single-ended configurations are small despite the very different flow patterns described by Vouriot *et al.* [12,13].

## 4. Conclusions

The distribution of a passive tracer representing infected breath was investigated using CFD to inform estimates of how environmental uncertainties, such as spatial variations in CO_${,}_{2}$_ and infected breath concentrations, might impact the prediction of far-field exposure and subsequent airborne infection risk modelling. The choice was made to model an idealized naturally ventilated classroom under steady-state conditions. The ventilation strategy was selected so that the environmental uncertainties evolved naturally with differing ventilation rates and flow patterns (as ventilation opening sizes, and their locations, were varied). Classrooms were selected as they are ubiquitous in education systems around the world and are typically densely occupied spaces with significant potential to contribute to the spread of airborne respiratory diseases. The location of the infected breath (mimicking an infectious occupant) was systematically varied and the relative exposure of potential susceptible occupants was examined as a function of their location within the room.

The specific set-up investigated was idealized by design so that the variations in far-field exposure due to the ventilation provision could be assessed independently. As such, convection was assumed to be the dominant mechanism for heat transfer and conductive and radiative effects were not explicitly modelled. Their inclusion is likely to affect the temperature distribution, the ventilation flow and thus the resulting exposure to infected breath; this is further detailed by Vouriot *et al.* [13]. Both the vent position and heat input were also idealized and further work is needed to determine how the results presented here will be affected by considering vertical vents, representing typical windows or doors, or multiple localized heat sources. In addition, since the focus was on far-field exposure, occupants’ breathing and the near-field were not explicitly modelled. Instead, their breath, along with the viral aerosols responsible for infection, were modelled as non-buoyant passive scalars with steady sources that cannot represent accurately the close range and might lead to conservative estimates of exposure.

Investigating the potential for far-field airborne exposure, relevant to infection risk of respiratory illnesses, we showed that the concentration of the infected breath scalar was most strongly dependent on the distance from the release location. This finding held true irrespective of the specific location of the infectious occupant; with regions closest to the infector typically exhibiting higher concentrations. However, the location of the infector and the flow patterns sometimes had an important secondary impact. For example, should an infector be within a stagnating area, infected breath had a tendency to become significantly more concentrated than if the source of infected breath was in the path of the ventilating flow.

The distribution of the infected breath scalar was then directly compared with the distribution of CO_${,}_{2}$_ representative of the breath of all occupants. When CO_${,}_{2}$_ measurements within an operational space are available, the risk of airborne infection is typically assessed from a limited number of point measurements that are used to determine the rebreathed fraction [1]. The far-field exposure ratio $R_{l}$ was used to assess the accuracy of such point measurements of CO_${,}_{2}$_ when predicting exposure to infected breath; it was shown that $R_{l}$ varies depending on the ventilation configuration and on the specific measurement location. However, throughout the majority of the breathing zone, the exposure predicted based on point measurements of CO_${,}_{2}$_ was found to accurately indicate—here, to within a factor of two—the actual exposure to infected breath within the simulations. This held true for all infected breath source locations investigated, as well as for all ventilation configurations and flow rates investigated (which exhibited significant differences in ventilation efficiencies and flow patterns, see [14]). Although the focus was on naturally ventilated spaces, the results are expected to hold for mechanically driven displacement ventilation. Furthermore, the implications of our findings for sensor placement were examined, determining that the potential for far-field exposure predicted using CO_${,}_{2}$_ measurements at, or near, walls had a similar level of uncertainty to the exposure determined elsewhere in the breathing zone.

Although this variation in the far-field exposure ratio $R_{l}$ due to environmental factors (by a factor of up to approximately two) can be regarded as significant in some contexts, the uncertainty introduced needs to be considered in the context of other uncertainties that are inherent when modelling airborne infection risk. For example, for a given respiratory illness, estimates of the appropriate quanta generation rate q can vary by around three orders of magnitude based on the choice of the activity level, the strain of the virus and other unspecified factors [22]; relative to this, uncertainties of a factor of two are of little consequence. Therefore, even in situations where the location of the infectious individual is unknown, as is typically the case, single point measurements of CO_${,}_{2}$_ still provide a useful tool to assess the potential for far-field exposure to infected breath, from which the airborne infection risk can be modelled.

## Data Availability

The data that support the findings of this work are openly available in ORDA at [23].
